# Pb(core)/ZnO(shell) nanowires obtained by microwave-assisted method

**DOI:** 10.1186/1556-276X-6-553

**Published:** 2011-10-10

**Authors:** F Solis-Pomar, MF Meléndrez, R Esparza, E Pérez-Tijerina

**Affiliations:** 1 Laboratorio de Nanociencias y Nanotecnología CICFiM (FCFM), Universidad Autónoma de Nuevo León, Monterrey, Nuevo León 66450, México; 2 Centro de Innovación, Investigación y Desarrollo en Ingeniería y Tecnología (CIIDIT) de la UANL-PIIT, Apodaca, Nuevo León 66600, México; 3 Department of Materials Engineering (DIMAT), Faculty of Engineering, 270 Edmundo Larenas, Casilla 160-C, University of Concepcion, Concepcion, Chile; 4 International Center for Nanotechnology and Advanced Materials, Department of Physics & Astronomy, University of Texas at San Antonio, One UTSA Circle, San Antonio, TX 78249, USA

## Abstract

In this study, Pb-filled ZnO nanowires [Pb(core)/ZnO(shell)] were synthesized by a simple and novel one-step vapor transport and condensation method by microwave-assisted decomposition of zinc ferrite. The synthesis was performed using a conventional oven at 1000 W and 5 min of treatment. After synthesis, a spongy white cotton-like material was obtained in the condensation zone of the reaction system. HRTEM analysis revealed that product consists of a Pb-(core) with (fcc) cubic structure that preferentially grows in the [111] direction and a hexagonal wurtzite ZnO-(Shell) that grows in the [001] direction. Nanowire length was more than 5 μm and a statistical analysis determined that the shell and core diameters were 21.00 ± 3.00 and 4.00 ± 1.00 nm, respectively. Experimental, structural details, and synthesis mechanism are discussed in this study.

## Introduction

One-dimensional (1D) nanostructures as wires, rods, belts, and tubes have attracted the attention of researchers because of their intrinsic properties and novel applications in various fields [1]. There are several nanostructured materials that are nowadays being widely used, these are based on those of zinc oxide which has excellent properties such as a wide energy band gap (3.37 eV) [2], a large exciton binding energy (60 meV) at room temperature [3], high optical gain (300 cm^-1^) [4], high mechanical and thermal stabilities [5], and radiation hardness [6]. Because of these properties, ZnO nanostructures have been applied as activated material in the electronic devices manufacture, e.g., gas sensors, nanoresonators, solar cells, waveguide, field emitters, nanocantilevers, nanolasers, transistors, and optoelectronic devices [7-14]. Furthermore, this material is very versatile to obtain several kinds of nanostructures, such as wires, rods, particles, belts, plates, tubes, and flowers, that have been synthesized through various methods. Among these are the chemical and physical methods, whose choice often depends on the type of application being sought. A recent interest in scientific research is the application of nanostructures as superconducting nanowires with diameters comparable to the superconducting coherence length that has served as a model system to study thermal and quantum phase slips [15]. In addition, these kinds of materials have a relatively high critical temperatures and stability at ambient atmosphere make them potential candidates for applications in other superconducting devices, for example, single photon detectors [16] and hot-electron bolometric mixers [17]. A nanostructured system with the application mentioned may be a core/shell nanostructure based on nanowires filled by a metal. If the core-metal has a suitable thermal expansion coefficient, then these materials [metal(core)/oxide(shell)]-type can also be used as nanosensors and nanothermometers. This is the reason why Ga/MgO, In/CNT, Ga/CNT, Au(Si)/Ga_2_O_3_, and Pb/CNT have been used as (core/shell)-NWs systems for the development of nanothermometers and superconducting nanosensors, being thus far the most sensitive nanothermometers those based on the thermal expansion of indium-filled carbon nanotubes (CNT). In summary, one can say that "the integration of superconducting and semiconductors nanostructures would be quite important for technological applications". Otherwise, among metal nanowires that have been obtained so far, Pb is a particularly attractive, important, and challenging target owing to its superconductivity and high reactivity. The synthesis process to obtain (core/shell)-NWs using Pb as core is not easy and usually a small portion of this nanostructure is obtained. For this reason, one-step CVD method and two-step template synthesis method have been developed to obtain those [18]. In this study, a novel, fast, and simple one-step method for the preparation of Pb(core)/ZnO(shell)-NWs based on the ZnFe_2_O_4 _decomposition assisted by microwave is detailed. As the starting material the mining industry process residue of the ZnO production was used and furthermore did not utilize any reagent or preferential growth precursor molecules. The synthesis presented in this study was reproducible and can be versatile to prepare several kinds of nanostructured materials using microwave-assisted decomposition of precursor salts or other industrial residue.

## Experiment

### Synthesis

**Figure 1:**
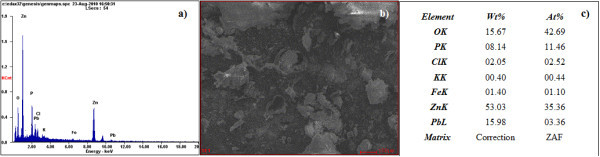
**SEM micrographs and EDX of the starting material**. **(a) **EDX and **(b) **SEM micrograph analysis of material used in the synthesis of Pb(core)/ZnO(shell) nanowires. **(c) **Composition analysis.

**Figure 2:**
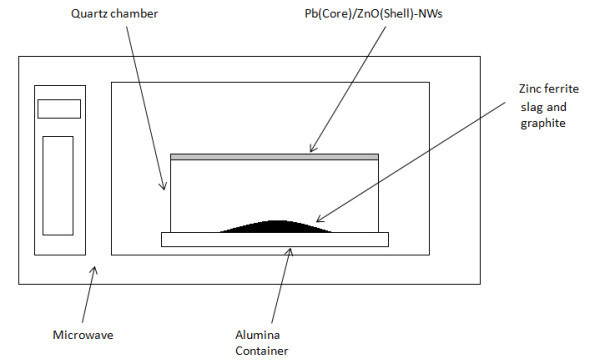
**Experimental setup**.

The starting material used for the synthesis was zinc ferrite slag contaminated with 3.35% of Pb (see Figure 1), purchased from Peñoles SA industrial service (México). A typical synthesis was carried out with zinc ferrite (1.0 g) mixed homogeneously with graphite (1.00 g). Evaporation procedure was performed using a conventional microwave oven (Sharp R658L (S)-Model) that was amended to do the experiments (see Figure 2). The reaction was carried out at 1000 W for 5 min. The product was condensed into a quartz chamber which extends from the material decomposition center to the upper surface of the microwave oven. The synthesized material was then collected at the top of condensation chamber.

### Characterization

High-resolution transmission electron microscopy was performed in a JEM-ARM200F probe aberration corrected analytical microscope with a resolution of 0.08 nm. Selected area electron diffraction was performed in a JEOL 2010F operating at 200 kV (point resolution of 0.19 nm). Scanning electron microscopy (SEM) was carried out using a FEG Hitachi S-5500 ultra high-resolution electron microscope (0.4 nm at 30 kV) with a BF/DF Duo-STEM detector and in a FEI-Nanonova 100 FE-SEM.

## Results and discussion

**Figure 3:**
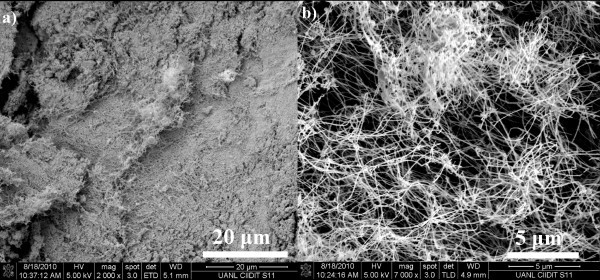
**SEM micrographs of Pb(core)/ZnO(shell) nanowires**. **(a) **Low magnification FE-SEM micrographs of Pb(core)/ZnO(shell) nanowires obtained by the decomposition-assisted microwave synthesis of zinc ferrite. **(b) **Nanowires seen at higher magnification. It is observed that the length of the nanowires is ~5 μm.

The synthesis process used in this study for the Pb(core)/ZnO(shell)-NWs preparation is simple, inexpensive, and was performed in solid state in the absence of chemical reagents and without preferential growth precursor molecule. After the synthesis, a spongy white material was obtained as cotton-like on the upper chamber condensate and the evaporation power always remained about 1000 W since below this value it was not possible to obtain the NWs. In addition, reaction time did not exceed more than 5 min since this was sufficient to complete reaction of the material and the graphite added to the reaction system which was to perform a reductive decomposition of the starting material and to facilitate the evaporation of the products. The SEM micrographs and EDX of the starting material used in this synthesis are shown in Figure 1; the EDX analysis shows that the slag also has other pollutants in lesser amount than the Pb such as K and Cl. The morphology analysis of the white powder obtained is shown in Figure 3, where the low-resolution FE-SEM micrographs of the NWs are shown. The technique effectiveness used to obtain the Pb(core)/ZnO(shell)-NWs is evidenced by the large percentage of the nanowires that are observed in the image. Furthermore, the conversion rate was over 90% and the material loss is only due to collection and cleaning processes of the material in the condensation chamber.

**Figure 4:**
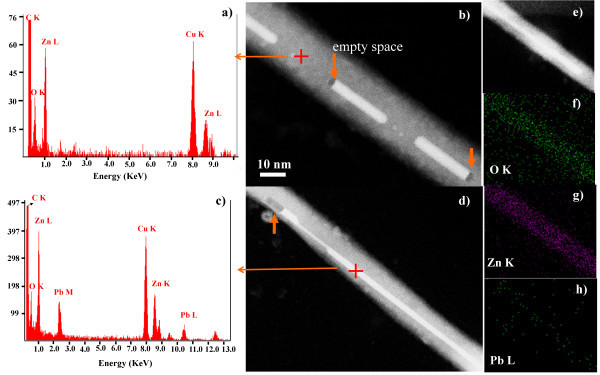
**EDX analysis and dark field HRTEM micrographs of Pb(core)/ZnO(shell) nanowires**. **(a, c) **EDX analysis of different NWs sections with and without Pb. **(b, d) **Dark field HRTEM micrographs where the analyzed sections in **(a, c) **are shown. **(e-h) **Mapping section of the Pb(core)/ZnO(shell)-NWs.

Figure 3a shows that the white powder obtained is composed mainly of ZnO nanowires whose length is up to 5 μm as can be seen in Figure 3b. The composition was determined by EDX point/line analysis and mapping, these results are shown in Figure 4. The EDX analysis and the dark field HRTEM micrographs of Pb/ZnO nanowires revealed the presence of Pb in the ZnO nanowires and also empty spaces within them as is shown in Figure 4b,d. These results are similar to those obtained by Wang et al. [19] who say that because of electron beam temperature the Pb-core expands moving from one extreme to another within ZnO-shell. Thermal expansion effect of partially Pb-filled nanotubes has also been studied by Lee et al. [20] who tested the electron beam effect on the Pb inner core of the nanotubes. Only a few studies have reported obtaining of Pb(core)/ZnO(shell) nanowires using wet chemical synthesis based on their respective nitrates [19]; however, the technique employed in this study consists of a one-step vapor transport, decomposition, and condensation reaction assisted by microwave to prepare these nanostructures is reported for the first time. On the other hand, it can be seen in the EDX spectra in Figure 4 that the Pb-core is within of the NWs owing to the signals appearing at 2.4 and 10.5 keV characteristics of Pb (M) and Pb (L), respectively. EDX analysis was performed on the brightest part of the NWs core and also in areas where there is no Pb (see Figure 4a,c); in these zones, the characteristics peaks of zinc [Zn (L); Zn (K)] and oxygen [O (K)] appear. The absence of Fe signals discards that the peaks of the spectra correspond to zinc ferrite. The maps of Zn (K), Pb (L) O (K) signals are shown in Figure 4e-h; these analyses confirmed that the NWs shell and core are formed of ZnO and Pb, respectively.

**Figure 5:**
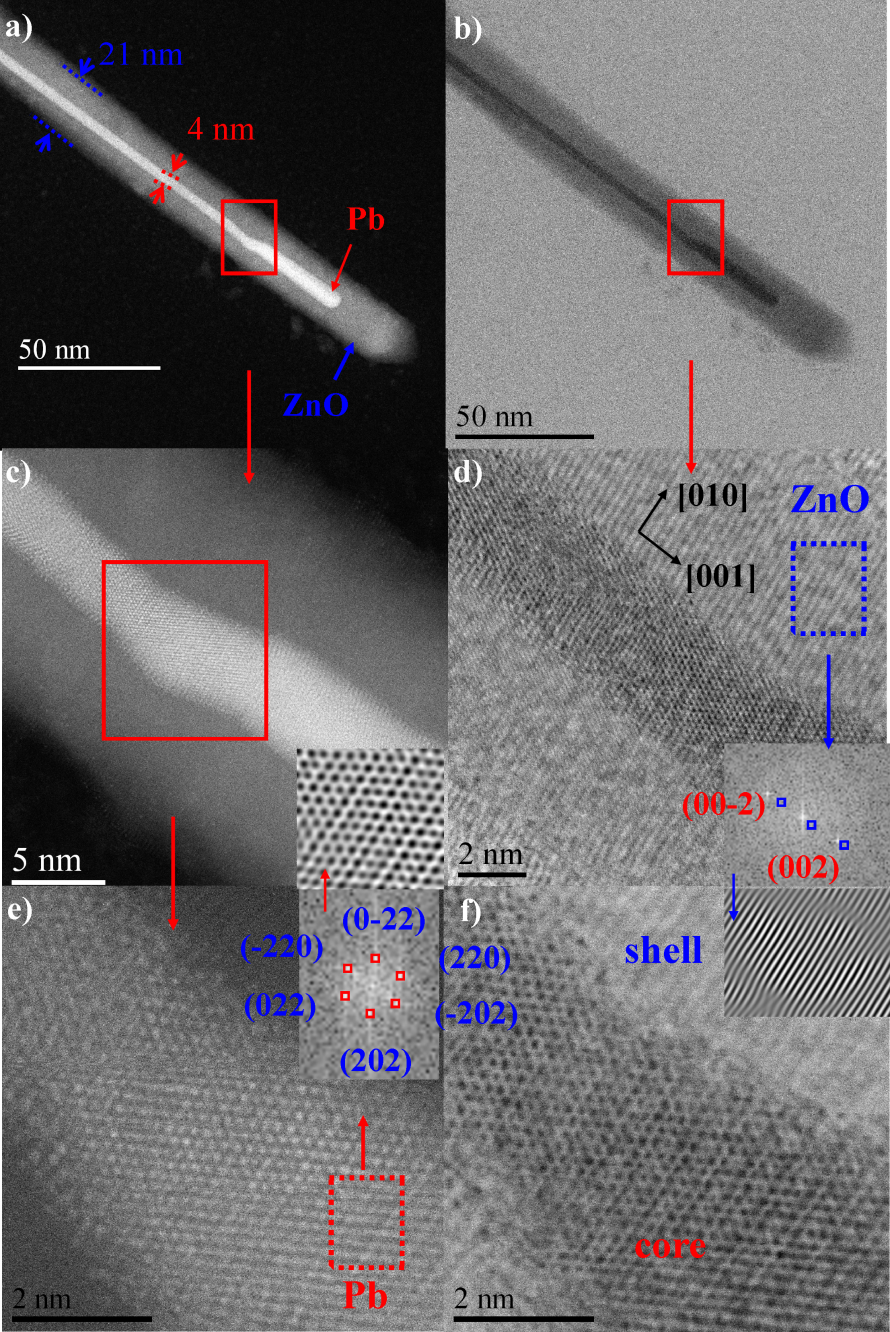
**Structural analysis Pb(core)/ZnO(shell) nanowires**. **(a, c, e) **HAADF-STEM micrograph and **(b, d f) **BF-STEM micrograph of the Pb(core)/ZnO(shell)-NWs. Internal diameter (Pb-core) is about 4 nm. **(d) **The ZnO-shell, the FFTs analysis confirms that the shell is ZnO hexagonal wurtzite-type with preferential growth in the [001] direction. **(e) **Pb can be seen; the inner wire core structure is Pb-cubic (fcc) with preferential growth in the [111] direction.

A more detailed structural analysis of the Pb-filled ZnO NWs is shown in the HRTEM micrographs in Figure 5c-f. From dark field micrographs in Figure 5a,c,e, one can clearly distinguish the Pb-core and ZnO-shell because of the differences in their atomic weights. It was determined through of the fast Fourier transform (FFTs) of the HRTEM image an interplanar distance (*d_hkl_*) of 1.75 Å characteristic of the {220} crystallographic planes of Pb with a cubic (fcc) structure. These results are in accordance with those reported by Wang et al. [19]. As mentioned, the Pb-core was highly susceptible to beam damage (especially for the relatively thinner region like the edge of a wire) when it was exposed to a flux of high-energy electrons. As a result, it is not unusual to observe that the right edge of this wire appears to have stacking defects, an artifact that might be caused by electron-beam-induced damage [18]. ZnO-shell analysis was performed from Figure 5d and it was determined that an interplanar distance (*d_hkl_*) of 2.60 Å corresponds to the (002) planes of the ZnO hexagonal wurtzite-type. In addition, from the HRTEM analysis it is determined that the zone axis crystal orientation is [100] and [001] which is the preferential growth direction characteristic of the ZnO nanostructures [21]. The NWs core and shell diameter were determined by random measurements and the obtained data were represented by a histogram; the average thickness was fitted to both normal and Gaussian curves. It was found that ZnO-shell and Pb-core thickness is around 21.00 ± 3.00 and 4.00 ± 1.00 nm, respectively, and the nanowires' length was ~5.00 μm. ZnO-shell thickness and length were independent of the microwave power used in the synthesis which ranged from 800 at 1000 W.

The proposed formation mechanism in this study is based on the ZnFe_2_O_4 _decomposition owing to temperature rise assisted by the microwave and also by Pb the evaporation process. Pb has a low melting point (327°C) and a boiling point of 1749°C so this just changes the state, melts, and evaporates. In the past, thermal decomposition of lead acetate in solid state has been studied by several groups and they conclude that depending on the temperature (up to 450°C) and environment (under N_2 _or in air), both Pb and PbO had been identified as the final products together with several types of basic lead acetate as the intermediates [22,23]. According to these studies, the minimum temperature required for the metallic lead production was around 325°C and it was found that lead could be formed as the major product (together with the formation of acetic acid as a byproduct). By contrast, in this study, the decomposition of zinc ferrite could be greatly facilitated because of the presence of hot spots, these spots are produced by the graphite in the mixture because it absorbs most of the incident radiation and acts as an evaporation source. Then there are two species in the vapor, the Pb evaporated from the slag and the ZnO produced of the ZnFe_2_O_4 _decomposition. The possible reactions are

(1) $$\text{ZnF}{\text{e}}_{2}{\text{O}}_{4}\left(\text{s}\right)+1∕2\text{C}{\text{O}}_{2}\left(\text{s}\right)\to 1∕2\text{C}{\text{O}}_{\text{2}}\left(\text{g}\right)+\text{Zn}\left(\text{g}\right)+\text{F}{\text{e}}_{2}{\text{O}}_{3}\left(\text{s}\right)$$

(2) $$\text{Zn}\left(\text{g}\right)+{\text{O}}_{2}\left(\text{g}\right)\to \text{Zno}\left(\text{g}\right)\Delta H=348.3\text{kJ}\;\text{mo}{\text{l}}^{-1}$$

(3) $$\text{ZnO}\left(\text{g}\right)\;\to \text{ZnO }\left(\text{s}\right)$$

(4) $$\text{Pb}\left(\text{s}\right)\to \text{Pb}\left(\text{g}\right)\;\Delta H=195.2\text{kJ}\;\text{mo}{\text{l}}^{-1}$$

(5) $$\text{Pb }\left(\text{g}\right)\to \text{ Pb }\left(\text{s}\right)$$

**Figure 6:**
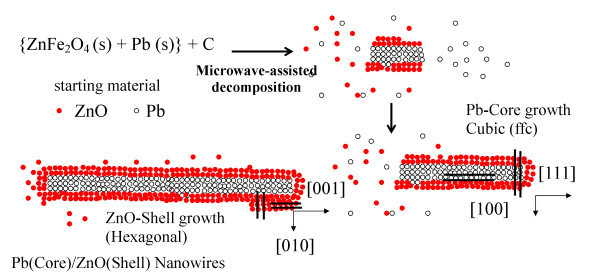
**Nucleation and growth mechanism of Pb(core)/ZnO(shell)-NWs obtained from the zinc ferrite slag contaminated with Pb through a decomposition reaction assisted by microwave radiation**. The reduction products are Fe (s), Fe_2_O_3 _(s), and CO_2 _(g). In the early stages of the reaction, the vapor is rich in Pb and has a ZnO lesser proportion. Some ZnO and Zn species react with the [100] Pb surface thereby facilitating its growth in the [111] direction.

In the initial stages, Pb(g) (Equations 4 and 5) will predominate and much less ZnO(g) and Fe_2_O_3 _(s) will be produced by virtue of their different heats of formation ZnO (348.3 kJ/mol) and Fe_2_O_3 _(824.2 kJ/mol). Thus, the formation of the Pb(core)/ZnO(shell)-NWs could be divided into two parts: (i) the microwave-assisted evaporation of Pb and (ii) the ZnFe_2_O_4 _decomposition. Therefore, Pb is in the vapor phase with some Zn and ZnO species and in the condensation process they interact stronger with the {100} facets of Pb that with the {111}. This selectivity can be attributed to the differences in the atoms configuration on these surfaces that may enhance or hinder their coordination to the molecules of ZnO and as a result, the side surface of a Pb nanowires (core) enclosed by {100} could be preferentially stabilized; while the ends, the {111} planes could be kept active and continues to grow (see Figure 6). Zinc oxide is produced in greater amounts than Pb, so once the Pb nanowires growth process is finished, ZnO continues to grow in the [001] direction until they coat completely the Pb and get the Pb(core)/ZnO(shell)-NWs as illustrated in the HRTEM micrographs.

## Conclusions

In this study, it was possible to obtain Pb(core)/ZnO(shell)-NWs using a slag of ZnFe_2_O_4 _with a Pb percentage of about 3% in the starting material, through a simple synthesis method which consisted of zinc ferrite decomposition assisted by microwave. This method proved to be versatile, economical, and reproducible to obtain this type of nanostructures. The nanowires core and shell had an inner and outer diameters of about 4 and 21 nm, respectively; with lengths greater than 5 μm. Length and thickness of both core and shell were independent of the radiation power used in the synthesis (800-1000 W). The nanowires core consisted of cubic Pb (fcc) with preferential growth in the [111] direction and the shell structure was ZnO hexagonal wurtzite-type. ZnO and Zn species interact more strongly with {100} facets of Pb facilitating its growth in the [111] direction.

## Competing interests

The authors declare that they have no competing interests.

## Authors' contributions

FSP carried out the experiment. MFM performed the results interpretation and drafted the manuscript. RE performed the characterization. EPT conceived of the study, design, and coordination. All the authors read and approved the final manuscript.
